# Continuously manufactured single-core iron oxide nanoparticles for cancer theranostics as valuable contribution in translational research

**DOI:** 10.1039/d0na00343c

**Published:** 2020-08-17

**Authors:** Regina Bleul, Abdulkader Baki, Christian Freese, Hendrik Paysen, Olaf Kosch, Frank Wiekhorst

**Affiliations:** Fraunhofer Institute for Microengineering and Microsystems (IMM) Carl-Zeiss-Strasse 18-20 55129 Mainz Germany regina.bleul@imm.fraunhofer.de; Physikalisch-Technische Bundesanstalt Abbestr. 2-12 10587 Berlin Germany

## Abstract

Micromixer technology was used to manufacture magnetic single core iron oxide nanoparticles that combine imaging as well as therapeutic functions. In a continuous, scalable and highly controllable manner, synthesis with biocompatible educts *via* an aqueous synthesis route was performed. Size control by varying relevant process parameters *e.g.* temperature was confirmed by transmission electron microscopy measurements of experimental series demonstrating the exceptional size control and homogeneity. Furthermore, analytical centrifugation evidenced the stably dispersed state of the single core nanoparticles in aqueous media. Size controlled production of single-core iron oxide nanoparticles was used to design optimized nanoparticles with a core diameter of about 30 nm, showing high signal amplitudes in Magnetic Particle Imaging (MPI) as a promising MPI tracer material. Moreover, therapeutic potential of these particles in magnetic fluid hyperthermia was evaluated and specific absorption rates (SAR values) up to 1 kW per g(Fe) were obtained, which exceed the comparable SAR value of Resovist® by more than a factor of three. Relaxometry measurements clearly confirmed the capacity of these single-core magnetic nanoparticles to generate significant *T*_2_-weighted magnetic resonance imaging (MRI) contrast that potentially allows multimodal imaging for monitoring the particles *in vivo* in a theranostic application scenario. Finally, first cell viability and apoptosis tests on endothelial cells did not show any cytotoxicity certifying a good biocompatibility of the iron oxide nanoparticles. This microtechnological approach provides reproducible, scalable single core iron oxide nanoparticles as highly performing tracers for MPI diagnosis as well as efficient heat generators for hyperthermia therapy. These preliminary results contribute to translational research in image guided cancer therapy – a further step from basic research to future medicine.

## Abstract 2

A micromixer platform for continuously manufactured single-core iron oxide nanoparticles for diagnostic and therapeutic applications contributing to translational research.

## Graphical abstract

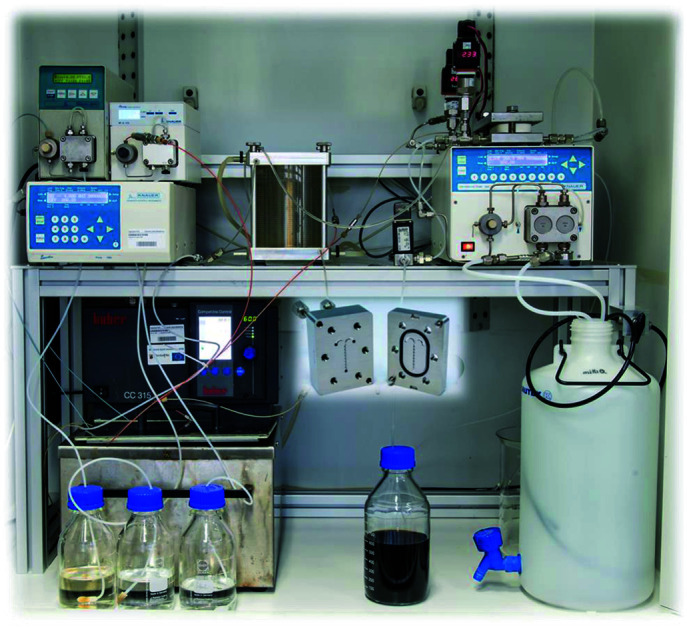

## Introduction

Cancer is still one of the leading causes of death worldwide, responsible for an estimated one million deaths in 2018. Even though many advances in both cancer diagnosis and cancer therapy have been achieved, aiming for early diagnosis and precise treatment at the right time and with appropriate dose, theranostic nanoparticles hold potential for revolutionizing future cancer treatment.^1–4^ There are numerous multifunctional nanosystems designed for a more specific and personalized disease management, which combine diagnostic and therapeutic capabilities in one single biocompatible (and biodegradable) nanoparticle.^1,5,6^

However, present theranostic nanoparticle approaches offering real-time cancer therapy monitoring are not in clinical practice yet. The translation of existing promising research approaches into clinical application often fails because of the disproportionally high complexity of sophisticated nanosystems raising huge issues already in reproducibly manufacturing sufficient amounts for comprehensive preclinical testing. Even for magnetic nanoparticle systems already tested in clinical trials, one study has been withdrawn due to high lot to lot variation of the nanoparticles.^7^

Magnetic particle imaging (MPI) is a novel imaging technology with great potential for cancer diagnosis using magnetic nanoparticles as a tracer material.^8,9^ MPI is in a preclinical state with further demand for improving both the MPI scanner infrastructure and the imaging performance of the tracer materials.^10^ Theoretical models suggest that single-core iron oxide nanoparticles of about 20–30 nm core diameter are optimal MPI tracers.^9,11–13^ Single-core iron oxide nanoparticles are of great interest not only for MPI, but also for the promising cancer treatment approach of magnetic fluid hyperthermia. Hyperthermia treatment using magnetic nanoparticles is already close to clinical practice particularly for brain tumours with further expanding indication fields *e.g.* prostate cancer.^14–16^ Theoretical estimations as well as experimental results of bacterial magnetosomes suggest huge potential of single core magnetite nanoparticles also in hyperthermia applications.^17,18^ Furthermore, cancer treatment with drug loaded magnetic carriers exploiting magnetic drug targeting as well as drug carriers co-loaded with imaging agents for drug monitoring are also of high-profile in the research landscape.^19–22^

Single-core particles with sizes larger than 20 nm are not easily accessible by standard synthesis methods like co-precipitation of iron salts. The technically more demanding thermal decomposition involves toxic educts and requires high temperature and phase transfer leading to a time-consuming multistep procedure.^23^ A promising alternative approach, the biotechnological production of single-core iron oxide nanoparticles, so-called magnetosomes from a bacterial origin, also suffers from effortful downstream processing, limited scalability, and potentially immunogenic residues inhibiting safe *in vivo* applications.^24^

Preliminary studies on the continuous synthesis of iron oxide nanoparticles demonstrated the high potential of micromixer technology as a valuable tool for the development of new magnetic nanomaterials.^25^ However, the performances of reported tracers for magnetic particle imaging at this time were still far below the performance of Resovist®, which is an MRI contrast agent and is presently also considered as a gold standard for MPI.

Here, we report high performance single-core iron oxide nanoparticles for versatile theranostic applications obtained from an enhanced microfluidics-based synthesis platform including downstream processing resulting in exceptional aqueous dispersion stability. With single-cores in the size range between 20 and 35 nm, they are within a comparable range to that of bacterial magnetosomes, but are advantageous concerning biocompatibility and scalability. With our platform technology, the tuneable, scalable production of size-controlled magnetic nanoparticles that can combine imaging (MPI and MRI) with therapeutic functions (hyperthermia, and drug loading) in one single nanoparticle system becomes feasible.

## Results and discussion

### Preparation of magnetic single-core nanoparticles

**Fig. 1:**
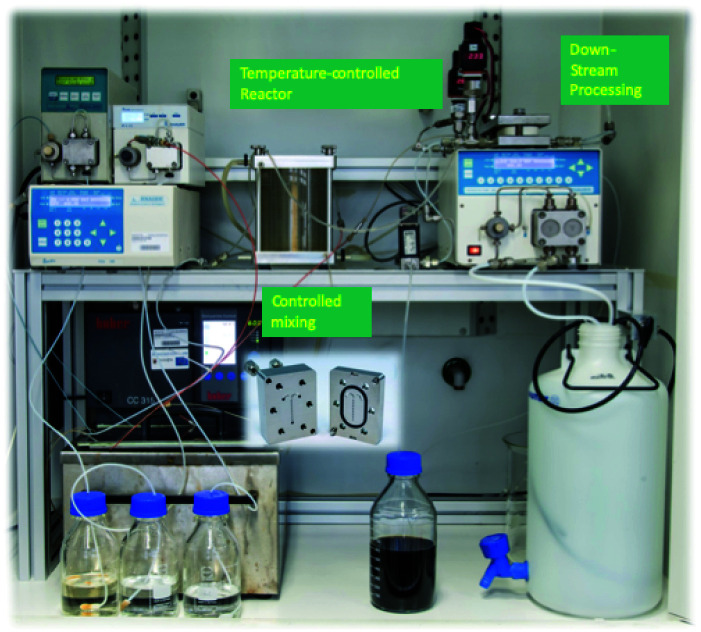
Photograph of the micromixer platform used for synthesis of single core iron-oxide MNPs.

Single-core magnetic iron oxide nanoparticles were manufactured continuously in a micromixer-based synthesis platform consisting of a nanoparticle generation module, and particle growth zone as well as a downstream processing module, as shown in the photograph in Fig. 1. Microfluidic systems for nanoparticle synthesis gained popularity in the last decade because of their promising potential in controlling critical stages such as nucleation and growth leading to improved size control, enhanced reproducibility and higher throughput than in batch synthesis.^26^ Even though microfluidic systems for magnetic nanoparticle systems have already been described, those devices are either limited by very low flow rates and throughputs, *e.g.* for droplet generation,^27^ or are based on thermal decomposition or supercritical organic solvents,^28–30^ which require high energy input (high temperature and pressure) and partially toxic agents. None of these approaches are single step procedures. Thus, additional steps for washing, stabilization and purification of nanoparticles are generally required leading to undesirable agglomeration and aggregation of initial single-core nanoparticles, which is particularly crucial for larger cores >15–20 nm.

This enhanced micromixer platform, based on a set-up already published previously,^25^ enables control over the particle size of single-core nanoparticles that are instantaneously coated and purified resulting in a ready-to-use stable aqueous nanoparticle dispersion.

To demonstrate the performance capacity of this modular manufacturing platform for the tuneable production of biocompatible iron oxide nanoparticles for versatile biomedical applications, a series of synthesis runs were performed. As a result, three single-core magnetic nanoparticle (MNP) systems T1, T2, and T3 with core size diameters in the range of 25, 30 and 35 nm were synthesized with analogous educt solutions and process parameters, solely by moderately varying the reaction temperature between 328 K and 338 K (Δ*T* = 5 K) far below the boiling point of water as one example of the relevant steering parameters. The higher the reaction temperature is, the faster the particles grow, so that in the same time interval keeping all other parameters such as the flow rate, dwell time, educts constant, particles with larger diameters are obtained. The structural and magnetic properties of these particles were characterized by Transmission Electron Microscopy (TEM) (core diameter), Differential Centrifugal Sedimentation (DCS) (hydrodynamic diameter), quasi-static DC Magnetization (DCM) (saturation magnetization), dynamic AC Susceptibility (ACS) (initial susceptibility) and Magnetic Particle Spectroscopy (MPS) (non-linear dynamic susceptibility). The relevant structural and magnetic parameters for the three samples are summarized in Table 1. Furthermore, experiments to demonstrate the performance of the systems as MPI tracers, hyperthermia agents and MRI contrast agents were carried out. In addition, we analysed the biocompatibility with first cytotoxicity and apoptosis tests on endothelial cells.

**Table 1:** Structural and magnetic parameters of continuously synthesized single-core iron oxide nanoparticle samples T1 (*d*_c_ = 23 nm), T2 (*d*_c_ = 30 nm), and T3 (*d*_c_ = 36 nm). For comparison, the corresponding literature values for Resovist® have been added. The values for *d*_c_ determined by TEM from Kraupner 2017;^39^*M*_S_ and magnetic diameters of the bimodal multicore system from Eberbeck 2011,^11^ MPS parameters 
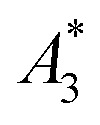
 and *A*_5_/*A*_3_ from Löwa 2017.^40^ Note, the number in parentheses behind the value denotes the variations of the parameters found in different *N* = 3–5 synthesis runs, *e.g.* 23(5) nm means 23 ± 5 nm. The parameter *σ* = *d*_c,SDV_/*d*_c,mean_ denotes the relative standard deviation of the size distribution determined by TEM

Syst	*d*_core_, nm	*σ*	*d*_hydr_, nm	*M*_S_, A m^2^ per kg(Fe)	*χ*_ini_, m^3^ per kg(Fe)	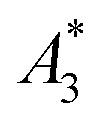 , A m^2^ per kg(Fe)	*A*_5_/*A*_3_, %
T1	23(5)	0.2(3)	21	90(5)	0.026(3)	13.5(8)	31.4(9)
T2	30(5)	0.16(2)	27.5	108(5)	0.055(3)	18.9(3)	33.4(3)
T3	36(6)	0.17(2)	35	101(6)	0.09(1)	16.1(8)	32(2)
Res.	6(3)		45(7)	90		8.67(3)	38.38(2)
	5						
	24						

### Structural and magnetic characterization of magnetic single-core nanoparticles

#### TEM and DCS results

**Fig. 2:**
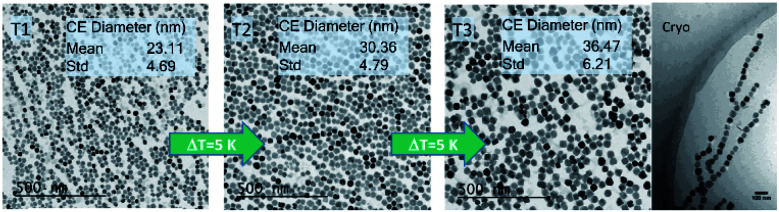
TEM images of single-core iron-oxide nanoparticle samples T1, T2, and T3 manufactured at increasing synthesis temperatures (Δ*T* = 5 K). Corresponding particle core diameters (mean and standard deviation; Std) obtained from the TEM image are shown in the inset. The cryogenic TEM picture shows the tendency of chain formation for particles with a core size larger than 30 nm (T3 sample).

**Fig. 3:**
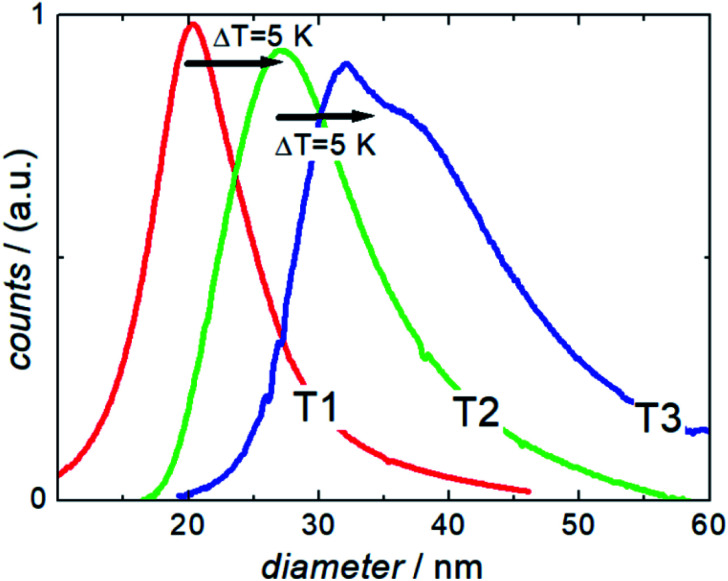
Analytical centrifugation (DCS measurements) of single core iron oxide nanoparticle samples T1, T2, and T3 synthesized at three different temperatures (Δ*T* = 5 K).

The TEM image shown in Fig. 2 clearly reveals the envisaged core size increase by increasing the reaction temperature.^25^ All three samples are mainly homogenous in morphology and size. A minor morphological shift from fully spherical shape for T1 (23 nm) synthesized at the lowest temperature to partly cubic particles for the larger particles T2 (30 nm) and T3 (36 nm) is visible. The MNPs produced with the micromixer platform are evidently single-core particles that have not formed any clusters or aggregates, which is often an unwanted effect for synthetic MNPs lacking sufficient stabilization. They show the same narrow size distribution with a relative standard deviation *σ* (ratio between the standard deviation and mean of the absolute diameter distribution as listed in the inset in Fig. 2) with *σ* ≤ 0.2 for all systems. To further evaluate dispersion stability and agglomeration status in aqueous dispersion, analytical centrifugation (DCS) was carried out. This analytical technique, which sensitively resolves the occurrence of aggregates and agglomerates, confirmed a relatively narrow distribution of hydrodynamic diameters without strong aggregation. In contrast to dynamic light scattering, where the high absorbance of the black magnetite particles interferes with the optical analysis of hydrodynamic diameters, DCS provides more reliable size information, as sedimentation within the disc centrifuge is proportional to the size as well as the density of the particle. The data in Fig. 3 show the density of pure magnetite neglecting the decrease in density by the organic layer on the surface of the particle. This leads to slight shifting of the hydrodynamic diameters determined by DCS to smaller values (as the real density of the particles is smaller than the assumed magnetite density used for the data analysis). This effect diminishes with increasing core sizes as the proportion of the organic layer decreases.

DCS measurements of the three samples showed the expected increase in hydrodynamic size with increasing synthesis temperature. Furthermore, they confirmed the exceptional dispersion stability of the relatively large magnetic cores in aqueous media. Compared to other synthesis methods, the presented one-step continuous process avoids additional precipitation and resuspension steps for subsequent stabilization or washing, thus undesirable clustering does rarely occur.

Nonetheless, the appearance of a peak shoulder for sample T3 clearly indicates the occurrence of particle–particle interactions due to its high magnetic moments and the onset of dimer, trimer, and chain formation. Tendency of reversible chain formation also in the absence of a magnetic field was observed already for particles with core diameters larger than 30 nm and confirmed by cryogenic electron microscopy as shown in Fig. 2 (right) for sample system T3.

The micromixer manufacturing platform enables access to single-core MNPs in the relevant size range for potential applications in nanomedicine (MPI and magnetic hyperthermia). Since not (solely) the physical size, but more importantly the magnetic properties determine the performance in biomedical magnetic applications, a further comprehensive characterization with multiple magnetic measurements was conducted.

#### Magnetic characterization

##### DCM results: saturation magnetization, and moment estimation

The saturation magnetization *M*_S_ of magnetic nanoparticles is a valuable magnetic parameter indicating the quality of the crystal structure and its homogeneity achieved by the nanoparticle synthesis process.

**Fig. 4:**
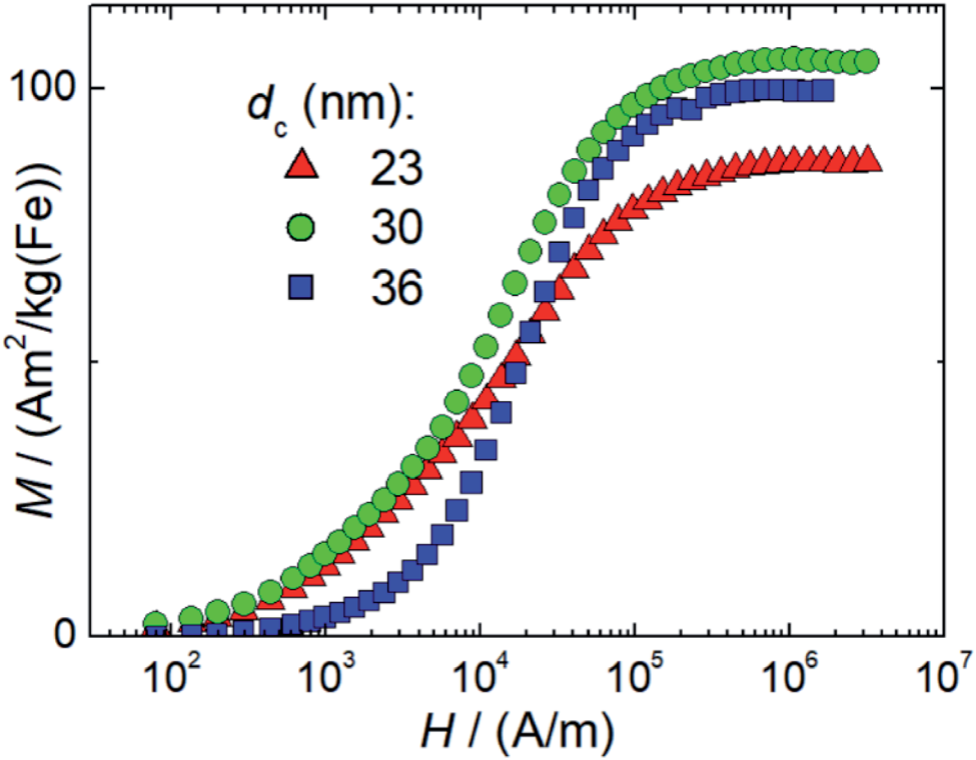
Room temperature (*T* = 295 K) DC magnetization curves of single-core iron oxide samples T1 (*d*_c_ = 23 nm), T2 (*d*_c_ = 30 nm), and T3 (*d*_c_ = 36 nm) synthesized at three different temperatures (Δ*T* = 5 K).

The room temperature magnetization curves for the three systems are shown in Fig. 4 from which the saturation magnetization *M*_S_ is determined. We observe *M*_S_-values above 108 A m^2^ per kg(Fe) for sample T2, 101 A m^2^ per kg(Fe) for sample T3, and 90 A m^2^ per kg(Fe) for sample T1, which is the same value that is found for Resovist®.^11^ All values are very close to *M*_S_ values (111–127 A m^2^ per kg(Fe)) reported for bulk magnetite or maghemite^31,32^ indicating the high crystallinity of the magnetite structure with a low amount of disorder reached by our synthesis.

The high *M*_S_-values of our systems, we suppose can be attributed to the presence of large proportions of highly magnetic magnetite and maghemite phases, which both have very similar magnetic properties. The structural composition of our nanoparticle systems regarding the occurrence of other non-magnetic hematite, and wustite and other iron oxide compounds such as iron hydroxides was not analysed. Moreover, other iron oxide nanoparticle systems also consist of a mixture of magnetite and maghemite, which are well tolerated by the body like the MRI contrast agent Resovist®.^33,34^ This reduction is attributed to crystallographic disorder or surface disorder caused by the coordination effects of organic ligands. The former leads to local disorder of the spin arrangement (locally) within the particle, and the latter at the particle surface, both reducing the coupled total particle moment *m*_p_ in a nanoparticle and thereby *M*_S_.

Generally, *M*_S_-values for magnetite (or maghemite) nanoparticles are observed to be below that of the pure bulk material.

A pronounced correlation between the particle size and *M*_S_ is reported in the small diameter range 1 nm to 10 nm due to the large surface-to-volume-ratio of these particles. The observed slight reduction of *M*_S_ in T1 with the smallest diameter might therefore be attributed to this effect while for the larger systems T2, and T3 the influence of surface disorder becomes insignificant. This behavior has been observed for multi-core nanoparticle systems, where several small sized nanoparticles are embedded in a polymer matrix to form a larger nanoparticle, so-called nanoflowers. The even increased surface-to-volume ratio in this system leads to strongly reduced *M*_S_-values of 60 A m^2^ per kg(Fe).^35^

Finally, at larger diameters as in sample T3 dipole–dipole interactions between the particles could lead to chain formation, which in turn would reduce the measured magnetization and thereby *M*_S_ of the particles.

For 30 nm single core MNP obtained by thermal decomposition Teeman *et al.* reported a *M*_S_-value of 96 A m^2^ per kg(Fe),^36^ which is close to the value of micromixer sample T2 (30 nm). In contrast to our aqueous synthesis approach, their laborious organic synthesis procedure requires a subsequent phase transfer step and takes about 40 h. Thus, not only concerning the presence of organic and toxic agents during the synthesis, but also regarding efficiency, our micromixer platform with residence times in the range of a few minutes represents an attractive alternative. From *M*_S_ = *m*_p_/*V*_p_ describing the ratio between the particle moment *m*_p_ and core volume *V*_c_ = π/6 6 *d*_c_^3^ as determined by TEM (see the TEM results above), we can estimate mean particle moments (=number of coupled individual atomic magnetic moments resulting in a single domain of total magnetic moment *m*_p_ within the nanoparticle) of 2 × 10^5^*μ*_B_ (T1), 6 × 10^5^*μ*_B_ (T2), and 10^6^*μ*_B_ (T3), respectively. Here, *μ*_B_ = 9.27 × 10^−24^ A m^2^ denotes the Bohr magneton. Preserving these large magnetic moments for the use in (biomedical) magnetic applications, thorough stabilization of single-core nanoparticle systems is required. To this end, the presence of the stabilizing agent already during the aqueous synthesis is a great advantage, as no additional precipitation step or phase transfer for postprocessing stabilization is required, which, due to the existing huge magnetic moments, would inevitably result in strong and inextricable agglomeration. This undesired effect was observed for single-core nanoparticles even below 30 nm, which formed agglomerates after phase transfer from thermal decomposition synthesis.^36^

The successful stabilization strategy in the micromixer synthesis is confirmed by DCM and ACS measurements. The DCM and ACS results for sample T1 (23 nm) as well as T2 (30 nm) show no agglomeration. A slight tendency is seen in sample T3 (36 nm) due to the initial formation of dimers, trimers or chains as confirmed by TEM. However, this chain formation is reversible as evidenced by DCM measurements after dilution and vigorous mixing of the samples.

##### Initial susceptibility, hydrodynamic diameter and stability

**Fig. 5:**
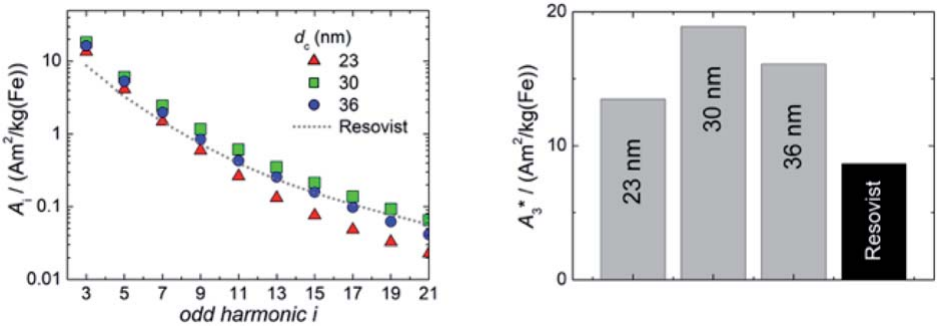
Non-linear dynamic magnetization spectra (left) measured at 25 kHz excitation frequency and 25 mT amplitude of single core iron oxide nanoparticle samples T1 (*d*_c_ = 23 nm), T2 (*d*_c_ = 30 nm), and T3 (*d*_c_ = 36 nm).

ACS measures the response of the magnetic moments *m*_p_ of the nanoparticles exposed to an alternating magnetic field. The moments will follow the excitation field but with a phase lag. Real (in-phase) *χ*′ and imaginary (out-of-phase) parts *χ*′′ of the complex dynamic mass susceptibility as a function of frequency *f* for the three systems are displayed in Fig. 5. The initial mass susceptibility *χ*_ini_ extracted from the extrapolation of *χ*′(*f*) for *f* → 0 increases with increasing core diameter from *χ*_ini_ = 0.026(3) m^3^ per kg(Fe) for T1 up to 0.09(1) m^3^ per kg(Fe) for T3. This is expected since *χ*_ini_ = (*M*_S_*V*_c_)^2^/(3*k*_B_*T*) = *m*_p_^2^/(3*k*_B_*T*) is proportional to the square of the particle moment, *e.g.* to the square of the core volume *V*_c_ of the particles. Furthermore, in the imaginary part *χ*′′(*f*) pronounced peaks with visible maxima at frequencies of 9.3(1) kHz (T1), 6.21(5) kHz (T2) and 2.8(1) kHz (T3) are observed. Following the Debye model,^37^ the excitation frequency *f*_p_ is at the peak position in the range of the inverse of the Brownian relaxation time *τ*_B_ = 3*V*_h_*η*/(3*k*_B_*T*) with *η* describing the viscosity of the medium of the nanoparticle with hydrodynamic volume *V*_h_. Therefore, the peak positions shift towards smaller frequencies for larger hydrodynamic diameters (*f_p_*(T3) < *f_p_*(T3) < *f_p_*(T1)) as a consequence that the larger particle moments already at lower frequencies cannot follow the sinusoidal excitation.

In T3 with the largest particle diameters the beginning of dipole–dipole interactions leading to chain formation or smaller aggregates (dimers, trimers) is attributed to the observed non-constant extrapolation of *χ*′ towards *f* = 0 and the broader maximum with a pronounced shoulder towards lower frequencies seen in *χ*′′(*f*).

##### Non-linear dynamic magnetic susceptibility

The non-linear dynamic magnetic susceptibility as measured by MPS is an important parameter to assess magnetic nanoparticle systems for their behaviour in both magnetic hyperthermia and MPI. Generally, single core nanoparticles with larger magnetic moments are favourable for magnetic hyperthermia and MPI.

**Fig. 6:**
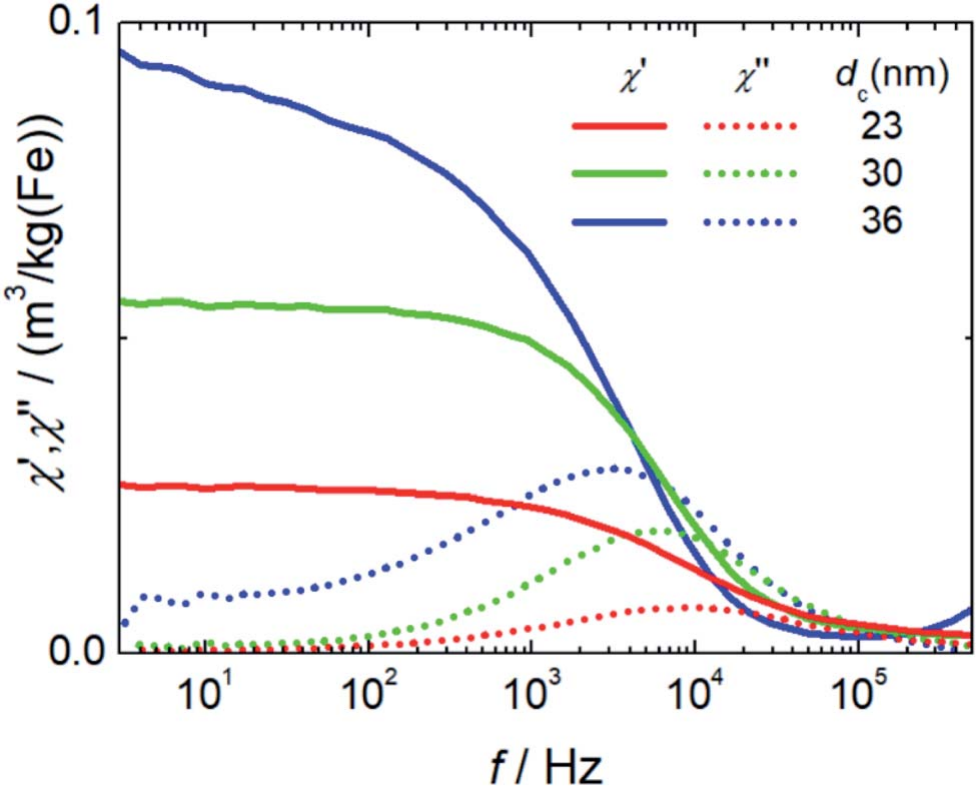
ACS measurements of single core iron oxide nanoparticle samples T1 (*d*_c_ = 23 nm), T2 (*d*_c_ = 30 nm), and T3 (*d*_c_ = 36 nm) synthesized at three different temperatures (Δ*T* = 5 K). The straight lines denote the real part, and the dotted lines the imaginary part of the complex dynamic susceptibility.

We determined the non-linear susceptibility by MPS at a fixed frequency (*f*_0_ = 25 kHz) and amplitude (*B*_excit_ = 25 mT). As shown in Fig. 6, all three systems exhibit non-linear susceptibilities (parametrized by 
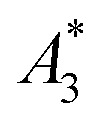
 in units A m^2^ per kg(Fe)) higher than that of the MPI gold standard and MRI contrast agent Resovist (
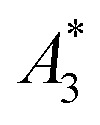
 = 9.1 A m^2^ per kg(Fe)) for T2 by nearly a factor of 2. These high 
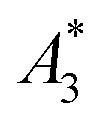
-values imply a high sensitivity, *e.g.* for spectroscopic applications like quantification of nanoparticles in cells and tissues by MPS.^38^ At higher harmonics *A*_i_, the dynamic susceptibility of Resovist® gets closer to the susceptibility of T2, indicating that in MPI, where the amplitudes of the higher harmonics *A*_i_ are important to ensure spatial resolution, both samples should exhibit equal image quality (see next section). After a storage time of 15 months at room temperature, MPS revealed for all 3 MNP systems minor relative changes below 5% in the MPS parameters 
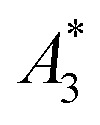
 and *A*_5_/*A*_3_ the high shelf life stability of the synthesis approach.

Relevant structural and magnetic parameters for the three MNP samples in comparison to Resovist® as a well characterized “gold standard” in the literature are presented in Table 1 (see below).

It is evident from the results presented so far that the properties of the single-core iron oxide magnetic nanoparticles synthesized by micromixer flow chemistry *via* an aqueous synthesis route can be tuned effectively by adjusting solely the temperature, the one relevant process parameter. Within a certain process window, the increase in temperature results in larger core diameters as observed by TEM. Moreover, the corresponding magnetic characterization confirms an increasing initial mass susceptibility *χ*_0_ that is proportional to the square of the particle moment. Larger magnetic cores lead to chain formation, which to some extent was observed structurally by analytical centrifugation as well as by cryogenic TEM and also confirmed magnetically by ACS measurements. These findings lead to estimated optimized particles of about 30 nm core diameters for application in MPI and magnetic hyperthermia, showing relatively high magnetic moments retaining dispersion stability.

## Performance of the nanoparticles in biomedical applications

### Diagnostic application: imaging capability of the nanoparticles

#### Magnetic particle imaging performance

Magnetic Particle Imaging (MPI) is an emerging tomographic imaging technology with simultaneous good spatial (mm) and excellent temporal (ms) resolution as well as high sensitivity where the image contrast is provided specifically by the magnetic nanoparticles without tissue background and without any need of ionizing radiation or radioactive tracers.^41^ The development and synthesis of tailored MPI contrast agents are an emerging and important actual research field.

**Fig. 7:**
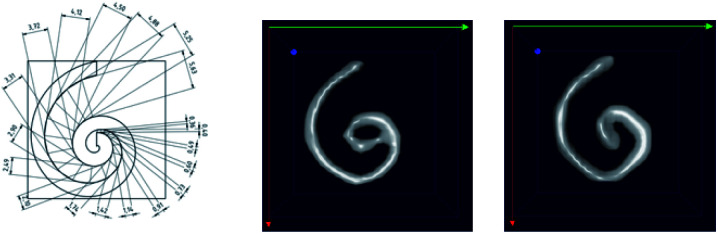
Spiral phantom geometry (left) and MPI images of the phantom filled with 200 μL volume 15 mM iron concentration of T2 (30 nm) (right) and Resovist® (middle). The channel width of the phantom was 2 mm.

MPI is based on the non-linear dynamic magnetic response of MNPs exposed to an alternating magnetic field, from which by using additional magnetic field gradients, an image of an MNP distribution can be obtained. To assess the potential of a nanoparticle system for MPI phantoms either MPS measurements – a kind of zero-dimensional MPI – of characteristic parameters 
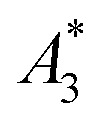
 and *A*_5_/*A*_3_ (see the section above) or direct MPI measurements of phantoms with a defined shape and nanoparticle concentration can be used. To exemplify the MPI imaging capabilities, Fig. 7 (middle) shows reconstructions of a spiral phantom filled with sample T2 (30 nm), which exhibits the highest non-linear susceptibility 
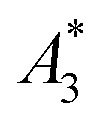
 measured by MPS. For comparison, we performed MPI measurements of the phantom filled with Resovist® (Fig. 7, right) at an identical iron concentration of 15 mM. Resovist®, originally a developed liver contrast agent for MRI, which is now extensively used as a tracer for MPI because of its good MPI performance.

The reconstruction of the MPI phantom is resolving mostly the complete spiral structure for both sample T2 as well as Resovist® with the same high quality.

From the border between the clearly resolved outer part of the spiral and the non-resolved inner central part of the spiral, a spatial resolution in the range of 1.5 mm was estimated for T2 and Resovist® for the MPI scanner settings, the particle concentration and within the uncertainty of the reconstruction.

With this behaviour, the single-core iron oxide sample T2 already now ranks among the high performance MPI tracers such as Resovist®. But there is space for additional fine-tuning of magnetic parameters to enhance the MPI performance not only by adjusting the size but also by addressing the internal magnetic structure through synthesis parameters of our micromixer approach. Due to the high flexibility, scalability and control of the micromixer technology it is an ideal tool to pursue this fine tuning of magnetic properties.

#### Magnetic resonance imaging contrast agent

**Fig. 8:**
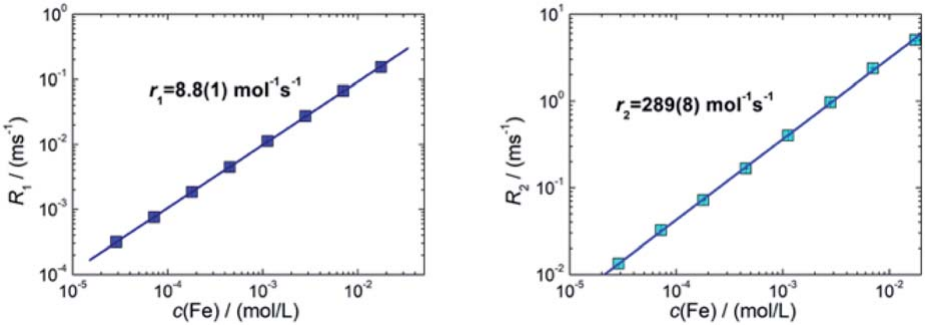
Room temperature NMR relaxation rates *R*_1_ (left) and *R*_2_ (right) as a function of iron concentration *c*(Fe) of sample T2 (30 nm) nanoparticles measured at 1.5 T. Note, for graphical representation the corresponding rates obtained by linear regression are displayed as straight lines.

The ability of iron oxide particles to increase the proton relaxation rates of the surrounding water proton spins makes them suitable as contrast agents in MRI and is described by the longitudinal *R*_1_ (=1/*T*_1_) and transversal relaxation rate *R*_2_ (=1/*T*_2_). This contrast improvement is based on stray fields caused and induced by the magnetic moments of the nanoparticles in the huge external *B*_0_ field acting on the proton relaxation in MRI. This is associated with the magnetic susceptibility of the particles as a function of particle size, composition and experimental variables, such as the magnetic field, strength of MRI, temperature and medium of dispersion.^42^ The relaxation rates as a function of iron concentration *c*(Fe) measured at *B*_0_ = 1.5 T are shown in Fig. 8 for sample T2 (30 nm) from which the MRI relaxivities have been determined and compared to those of the clinically approved MRI liver contrast agent Resovist® (multi-core nanoparticles with bimodal size distribution clusters of *d* = 5 nm and 24 nm).^11^

For sample T2 (30 nm) we determined relaxivities of *r*_1_ = 8.8(1) L mol^−1^ s^−1^ and *r*_2_ = 289(8) L mol^−1^ s^−1^. For T3 (35 nm) we measured *r*_1_ = 2.9(1) L mol^−1^ s^−1^ and *r*_2_ = 59(1) L mol^−1^ s^−1^. For the MRI liver contrast agent Resovist® values of *r*_1_ = 7.4 L mol^−1^ s^−1^ and *r*_2_ = 95 L mol^−1^ s^−1^ are reported in the literature at the same field *B*_0_ = 1.5 T.^43^ The relaxivities for these systems are shown in Table 2. We found a three times higher *r*_2_-relaxivity for T2 compared to Resovist®. MNP with high transverse relaxivity *r*_2_ can be used as an effective negative contrast agent. This feature is associated with the magnetic susceptibility and crucially depends on the particle size, composition and experimental variables such as the magnetic field, strength of MRI, temperature and medium of dispersion.^42^ In addition to a high *r*_2_ relaxivity, the efficiency of a T_2_ contrast agent relies on the ratio *r*_2_/*r*_1_ between transversal and longitudinal relaxivities.^44^ We find a specific enhancement of this ratio in both samples in comparison to Resovist®. Our results indicate that already sample T3, but moreover sample T2 exhibits a better contrast in *T*_2_-weighted MR imaging than Resovist®. Since Resovist® has been withdrawn from the market,^45^ our synthesis approach would be useful to develop an alternative iron oxide MRI contrast agent like T2 that would yield even better image quality.

**Table 2:** NMR *r*_1_- and *r*_2_-relaxivities at *B*_0_ = 1.5 T of single-core sample T2 (30 nm), and T3 (36 nm) together with values taken from Modo 2007 (ref. 43) for the liver contrast agent Resovist® (multi-core system, and bimodal size distribution)

System	*d*_c_, nm	*B*_0_, T	*r*_1_, L mol^−1^ s^−1^	*r*_2_, L mol^−1^ s^−1^	*r*_2_/*r*_1_
T2	30	1.5	8.8(1)	289(8)	33
T3	35	1.5	2.9(1)	59(1)	20
Resovist® (multi-core)	5, 24	1.5	7.4	95	13

### Therapeutic application: magnetic fluid hyperthermia (MFH)

Magnetic fluid hyperthermia is a potential technique for cancer therapy that exploits heat generated by magnetic nanoparticles exposed to a sinusoidal alternating magnetic field to kill cancerous cells.^46^ Many studies have shown that MFH is effective in killing cancer cells both *in vitro* and *in vivo* and that MFH crucially depends on the magnetic properties of the nanoparticles, the excitation frequency and amplitude of the alternating field to generate heat. The specific absorption rate (SAR), sometimes also denoted as specific loss power, is an important parameter to assess the capability of a magnetic nanoparticle system for MFH. The SAR value defined as the power dissipated into heat per unit of mass of nanoparticles, crucially depends on external parameters such as frequency *f* and amplitude *H* of the excitation field, as well as on internal nanoparticle characteristics. Here, structural factors such as the core size, size distribution, particle shape and crystallinity as well as magnetic properties such as saturation magnetization, anisotropy, relaxation time, concentrations and particle–particle interactions are important.^47^ To become independent of the external parameters *f* and *H*, the intrinsic loss power defined as ILP = SAR/(*H*^2^ × *f*) in units of nH m^2^ per kg(Fe) is useful since it allows the system-independent direct comparisons between experiments performed at different frequencies or amplitudes.

**Fig. 9:**
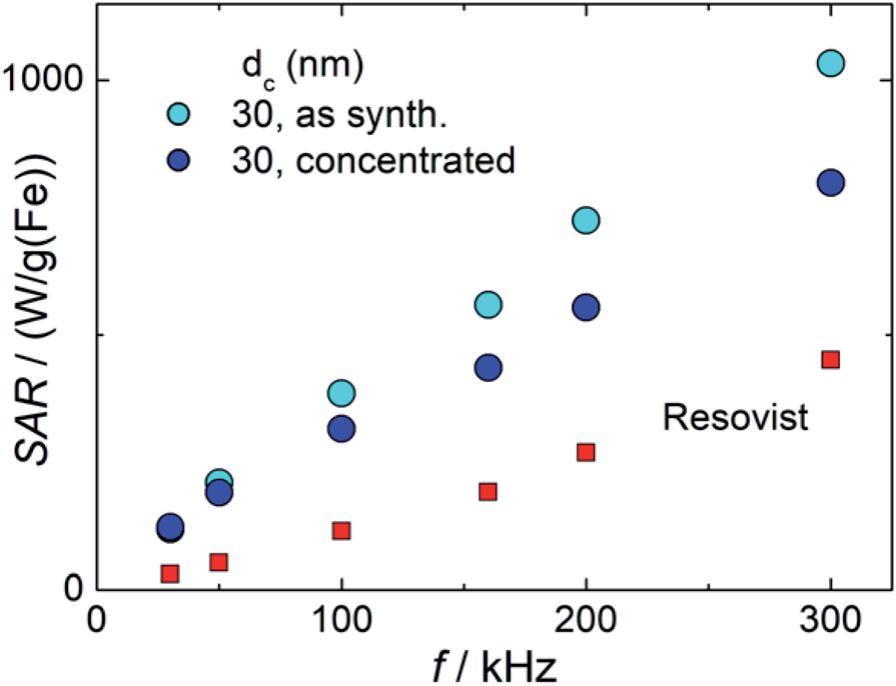
Measured SAR values *versus* the frequency of single-core iron-oxide nanoparticle system T2 (30 nm) as synthesized (cyan circles), and concentrated (blue circles) and of the MRI contrast agent Resovist®.

Fig. 9 shows the measured SAR values as a function of the frequency of sample T2 in two different formulations (as synthesized and concentrated) in comparison with Resovist®. All systems show the expected linear increase of SAR with frequency, while both T2 systems exhibit a much steeper slope compared to Resovist®. From the SAR values, corresponding ILP values for T2 (as synthesized) of 9.4(7) nH m^2^ per kg(Fe), and for T2 (concentrated) of 8(1) nH m^2^ per kg(Fe) were calculated. The values of T2 are much higher than typical ILP values found in the literature,^48^ which are in the range of 0.2–5 nH m^2^ per kg(Fe) (like the ILP = 3.0(5) nH m^2^ per kg(Fe) measured for our Resovist® sample). These remarkably high ILP values for the T2 system documents the excellent hyperthermia capability of the single-core particles obtained by our micromixer synthesis platform.

From a clinical point of view, higher SAR (or ILP) values are beneficial since these allow lower nanoparticle doses to achieve the same hyperthermia efficacy. It is therefore important to further understand and optimize the parameters that affect heat dissipation of MFH, such as particle diameters, structures, and surface coating. A comprehensive understanding of the physics of the relevant effects employed in both imaging and magnetic hyperthermia is related to the structural and above all magnetic properties. This includes the effective saturation magnetization *M*_S_, and the (dynamic) magnetic moment relaxation, as well as particle sizes and surface characteristics. Additionally, the impact of the dispersion and surrounding medium as well as changes in the physiological environment (like viscosity, and pH values) has to be considered. Although there are many accepted models aiming to simulate the time-dependent magnetic response as a function of an applied magnetic field, there are no simple analytical solutions to directly obtain the synthesis parameters from which magnetic nanoparticles with maximum performance in magnetic hyperthermia and imaging could be manufactured. Therefore, a synthesis approach with accurate control of individual nanoparticle properties is highly valuable, not only for the development of nanoparticle systems with increased hyperthermia and imaging performance but also for numerical model validation describing the complex physics of magnetic nanoparticles in biological environments. Our micromixer-based synthesis platform producing single-core iron oxide nanoparticles is a valuable tool to support this research.

### Biocompatibility evaluation in a cell culture

For all biomedical applications and particularly for future *in vivo* applications *e.g.* as imaging agents, hyperthermia agents or drug carriers in therapy, safety considerations are of significant importance. At this point we performed a preliminary cell culture cell viability study as a basic requisite for further preclinical investigations. Human Cardiac Microvascular Endothelial Cells (hCMEC) were used to assess cytotoxic effects of the synthesized nanoparticles.

Cell viability was investigated using the cellular deoxygenase assay, the WST-8 test with a cell counting kit-8 (CCK-8). Photometrical analysis of the amount of formazan produced by the cells allow the evaluation of the cell viability as it is directly proportional to the number of living cells. Since the CCK-8 solution is very stable, longer incubation periods, such as 24 to 72 hours, are also feasible. Moreover, the detection sensitivity is higher than that of of any other tetrazolium salts such as MTT, XTT or MTS. Furthermore, formazan dye is released in the medium and is analysed in a cell free supernatant. Thus, interference of the absorbance of internalized nanoparticles is negligible.

**Fig. 10:**
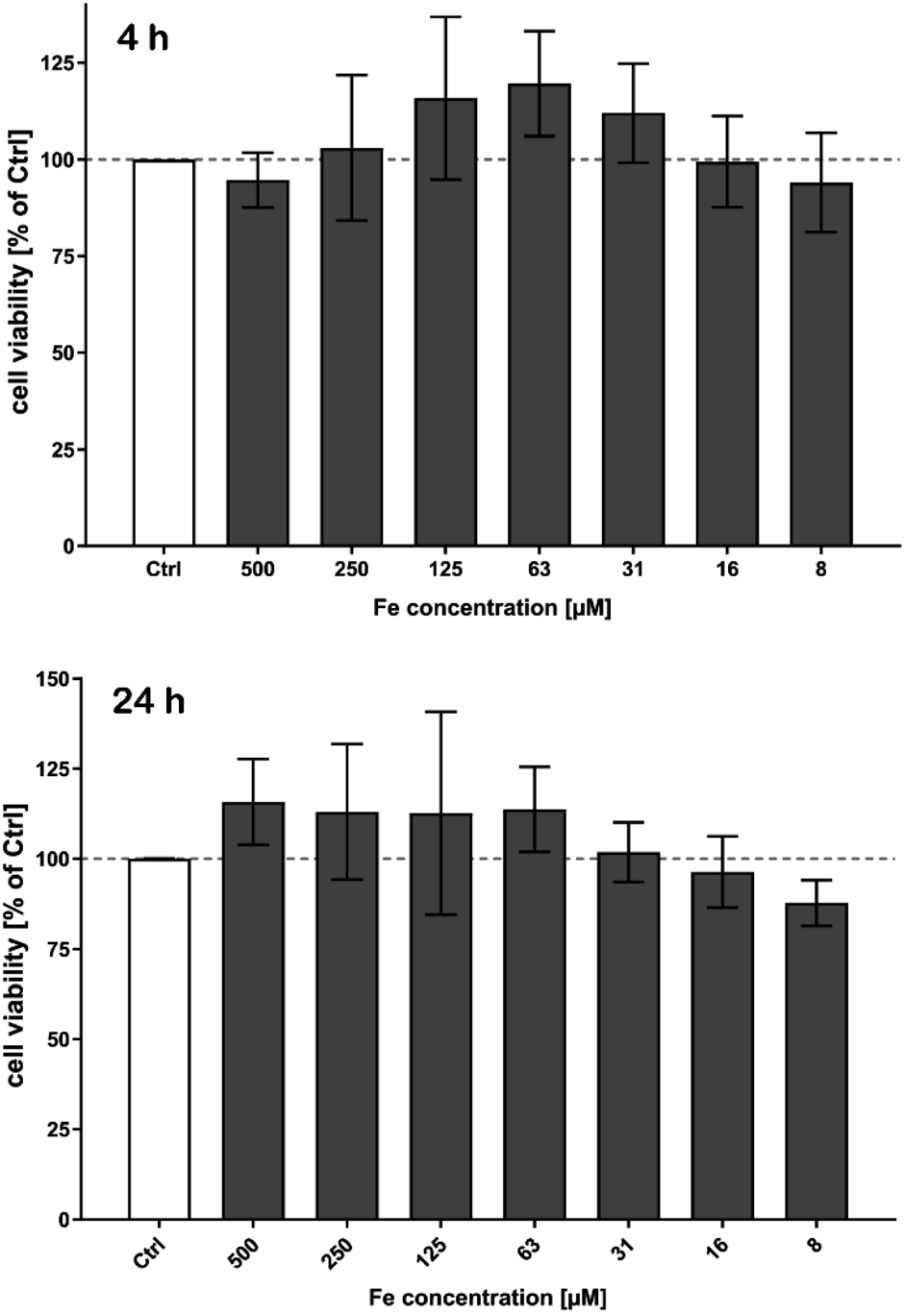
Determination of cell viability of brain microvascular endothelial cells (hCMEC) after treatment with MNP samples (exemplarily T1). Cells were seeded on 96-well plates and treated with several concentrations of nanoparticles for various time periods. Cells were washed and thereafter incubated with a CCK-8 substrate. After 60 minutes, the absorbance was measured at *λ* = 450 nm. Untreated cells (Ctrl) were set to 100% viability. No cytotoxic effects were observed neither short term (4 h) (top) nor long-term up to 72 h (bottom).

No cytotoxic effects on cell viability of brain endothelial cells after treatment with MNP samples in the investigated concentration range were observed within the detection limits neither short-term (4 h), see Fig. 10, nor long term up to 72 h.

**Fig. 11:**
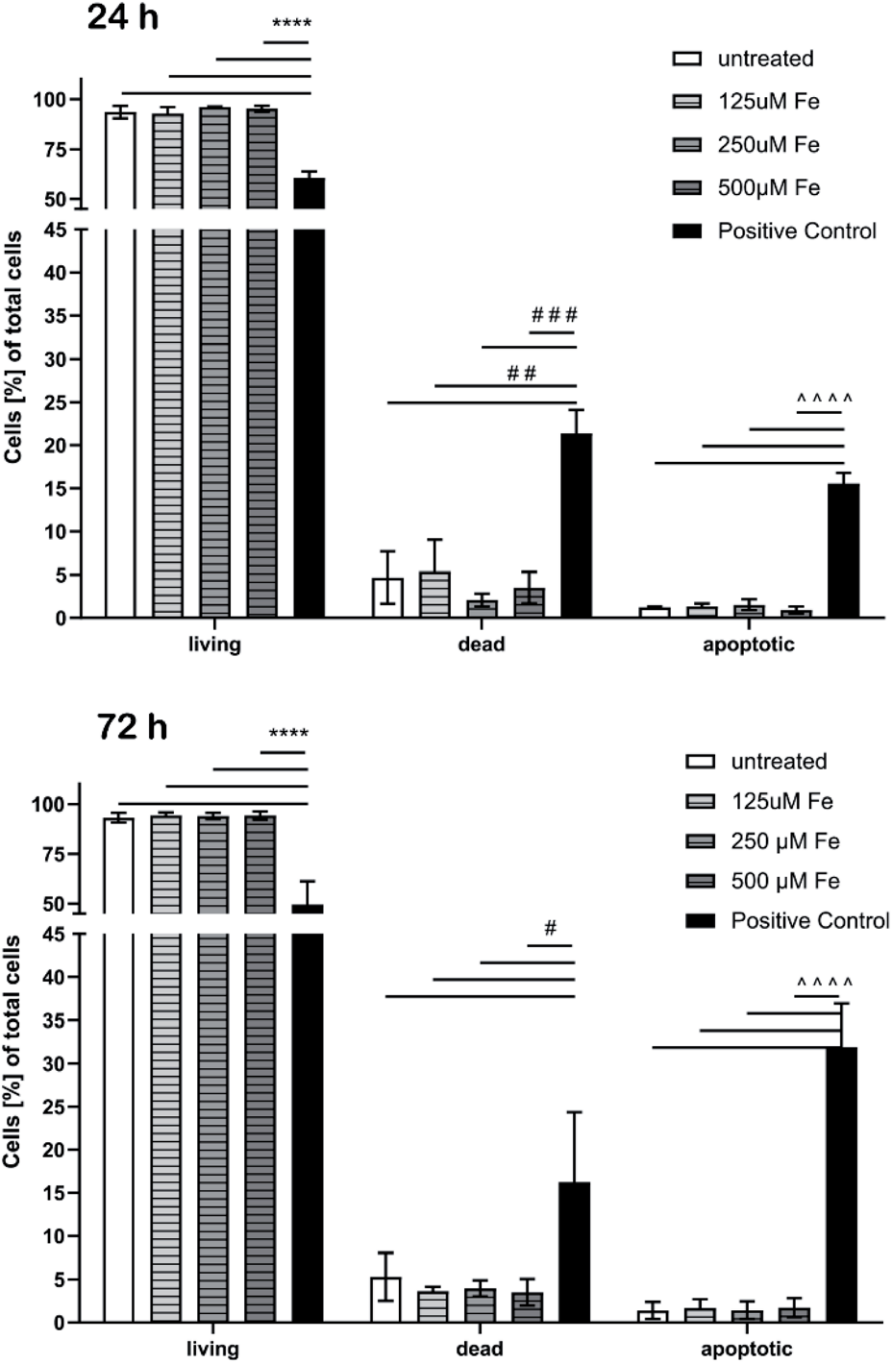
Determination of cell death and apoptosis of brain microvascular endothelial cells after treatment with MNPs for 24 h (top) and 72 h (bottom). Cells were analyzed by flow cytometry after staining with annexin-V-FITC and propidium iodide. Cell populations in percent of total cells have been blotted. One-way ANOVA followed by Tukey's multiple comparisons test was performed. The data of each group (living, dead, and apoptotic) were statistically evaluated separately from each other (*n* = 3–4).

Besides the cell viability, apoptosis caused by MNP incubation was studied. We used an annexin V-FITC kit that allows fluorescence detection of annexin V bound to apoptotic cells and enables the quantitative determination by flow cytometry. Propidium iodide (PI) was used to label the cellular DNA in necrotic cells with comprised cell membrane. Thus, differentiation among early apoptotic cells (annexin V positive and PI negative), necrotic cells (annexin V positive and PI positive), and viable cells (annexin V negative and PI negative) was possible. The apoptosis test also did not show any significant cytotoxic effects after 24 h and 72 h (Fig. 11), respectively.

The proportion of apoptotic cells of the MNP incubated samples was below 2% while DMSO used as a positive control showed drastic effects and confirmed the reliability of the test.

This preliminary cell culture study with endothelial cells lining the blood vasculature confirms the expected biocompatibility of the continuously manufactured iron oxide single-core nanoparticles. The MNP production is based on an aqueous synthesis route with non-toxic agents or organic solvents. Thus, basic requirements for application in biomedical (*in vivo*) applications are fulfilled. Further studies on size-effects of MNPs are ongoing.

## Experimental

### Synthesis details

Single-core iron oxide nanoparticles were synthesised by precipitation from aqueous, alkaline solutions of iron salts based on a micromixer set-up as previously reported.^25^ The microfluidic synthesis platform consists of HPLC pumps (Knauer, Germany), a caterpillar micromixer (Fraunhofer IMM, Germany) to induce particle nucleation and several temperature-controlled reaction loops to control particle growth and was enhanced by a downstream-processing-module to remove reactive agents and excess of stabilizing agents. Briefly, solutions of iron chloride, sodium nitrate as the oxidizing agent and sodium hydroxide were mixed in a caterpillar micromixer (Fraunhofer IMM) with symmetric liquid ratios and piped in a temperature-controlled reaction loop. Final nanoparticle dispersions were prevented from further oxidation as well as agglomeration with the addition of tannic acid (1.7 kDa) as the stabilizing agent. Finally, nanoparticles were purified by removal of unreacted educts and accessing of the stabilizing agent *via* diafiltration and magnetic separation and stored at room temperature for further analysis. In the present study, the size of magnetic nanoparticles was adjusted by varying only one process parameter: the reaction temperature from 328 K to 338 K (Δ*T* = 5 K). All other parameters including educt solutions, mixing ratios and flow rates were kept constant. Using a precise heat control (Huber thermostatic bath) and thin-walled tubes (thickness < 0.8 mm) for good thermal contact, a temperature stability below 1.5 K could be achieved. With our laboratory setup a temperature and reaction time dependent production yield in the range 20–50% can be achieved. Within 3 hours, a total volume of more than 4 liters can be provided. By adjusting the size of the microfluidic mixer (internal scale up) or by using several mixers in parallel (parallelization scale-up), the throughput can be increased easily by a factor of 15. The reproducibility was observed to be mainly determined by the variation of the chemicals, *e.g.* iron chloride batches while only little variation, <3%, (in the magnetic parameters as determined by MPS) is observed in the reproduction of samples using the same batch of chemicals.

For the evaluation of hyperthermia performance, particle dispersion was concentrated (Eppendorf Concentrator plus) up to 280 mM final iron content.

### Structural characterization of magnetic single-core nanoparticles

#### Transmission electron microscopy (TEM)

Transmission Electron Microscopy (TEM) measurements of nanoparticles droplet dried on carbon coated copper grids were performed with a Zeiss Libra 120 electron microscope at 120 kV acceleration voltage. A magnetic field was applied to the grids for a short period of time (about 10 minutes). The images were obtained using a CCD camera and from a selection of 1000–2000 individual nanoparticles the mean diameter and standard deviation of the core diameter were determined automatically using the open source software ImageJ.

For Cryo-TEM measurements, the same device at 120 kV acceleration voltage was used under liquid N_2_ conditions. Samples were prepared by applying a 6 μL drop to a cleaned carbon-coated copper grid, blotting with filter paper, and immediately proceeding with vitrification in liquid ethane at −180 °C. Grids were stored under liquid nitrogen until being transferred to an electron microscope for imaging.

#### Analytical centrifugation (DCS)

An ensemble method to investigate the particle dispersion in aqueous media is analytical centrifugation (Differential Centrifugal Sedimentation, DCS). DCS measurements were performed at 20 000 rpm (21 504 rcf) (CPS Instruments Inc. Measurements) after calibration with a silicon dioxide standard (255 nm). A sucrose gradient was built using 24% to 8% sucrose. The peak maximum was evaluated using Origin® software.

### Magnetic characterization of magnetic single-core nanoparticles

#### DC magnetization measurements (DCM)

Room temperature (*T* = 295 K) DC magnetization measurements (DCM) of MNPs were performed using a SQUID magnetometer (MPMS-XL, Quantum Design, USA). The device measures the magnetic moment *m*(*H*_e_) of a 30 μL sample volume (immobilized in mannitol to prevent chain formation or aggregation effects during the measurements) as a function of an external magnetic field *H*_e_ up to 4 × 10^6^ A m^−1^ (*B* = 5 T). The (mass) magnetization *M*(*H*) (in units A m^2^ per kg(Fe)) is obtained by normalizing to the total iron amount of the sample and the saturation magnetization *M*_S_ = *M*|_*B*=5 T_ was determined after subtracting a linear (paramagnetic or diamagnetic) background contribution. An overall measurement uncertainty of about 2.5% with 1% contribution from the measurement device and 2% due to preparation and the iron concentration determination is estimated. We did not perform any fitting of *M*(*H*) curves using the common Langevin model because the assumptions of isotropy (no crystal anisotropy) and thermal equilibrium of this model at room temperature become invalid for diameters larger than 20 nm.

#### Linear dynamic susceptibility measurements (ACS)

Room temperature (*T* = 295 K) linear magnetic AC susceptibility (ACS) of MNPs was measured with a commercial AC susceptometer (DynoMag, RISE Acreo, Sweden). For the measurements, a quartz glass cuvette was filled with a sample volume of 100 μL MNP suspension and the real *χ*′(*f*) and imaginary *χ*′′(*f*) parts of magnetic susceptibility were acquired in the frequency range 1 Hz to 100 kHz at an excitation amplitude of 0.2 mT. The initial mass susceptibility *χ*_0_ (in units m^3^ per kg(Fe), normalized to the sample iron mass) was obtained by extrapolation of the real part susceptibility*χ*′(*f*)|_*f*→0_.

#### Non-linear dynamic susceptibility measurements (MPS)

The non-linear dynamic AC susceptibility of MNPs is an important magnetic parameter for magnetic hyperthermia and magnetic particle imaging. Measurements were performed at *T* = 37 °C using a magnetic particle spectrometer (MPS) on a commercial spectrometer (MPS-3, Bruker, Germany) operating at an amplitude *B*_excit_ = 25 mT and fixed frequency *f*_0_ = 25 kHz. For the measurements a fast reaction tube (Applied Biosystems®, MicroAmp) containing a sample volume of 30 μL was placed in the detection coil of the MPS system. After Fourier transform of the detected time signal, the spectral components *A*_i_ of the non-linear AC susceptibility were obtained showing distinctive amplitudes at odd multiples (harmonics) of the excitation frequency *n* li*f*_0_, *n* = 3, 5, 7, …. Two characteristic parameters are extracted from the harmonic spectra, the amplitude of the third harmonic normalized to the iron amount of the sample, 
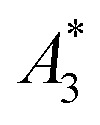
 (in units A m^2^ per kg(Fe)), and the concentration independent ratio between 5th and 3rd harmonic, *A*_5_/*A*_3_ (in units %). Both are correlated with the MPI performance with the general observation that the higher the 
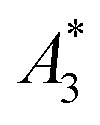
, and *A*_5_/*A*_3,_ the better the MPI images. To assess the shelf life stability of our samples, we remeasured sample aliquots of the stock suspension after 15 months of storage at room temperature and determined the relative changes in 
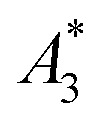
, and *A*_5_/*A*_3_.

#### NMR relaxivities (*r*_1_ and *r*_2_)

MRI properties were investigated by measuring longitudinal *T*_1_ and transversal *T*_2_ protons relaxation times for selected nanoparticle systems of 200 μL volume diluted to different iron concentrations *c*(Fe). The relaxation time measurements were carried out in a Minispec mq60 (Bruker) at *T* = 37 °C and a magnetic field of 1.5 T. For *T*_1_ a 2-pulse inversion-recovery sequence with a fixed relaxation delay of at least 5*T*_1_ was used, and *T*_2_ was determined employing a Carr–Purcell–Meiboom–Gill sequence, which consists of a 90° pulse followed by a series of 180° pulses, ideally covering the full decay of the signal. From the graphs of the iron-concentration *c*(Fe) dependent relaxation times *R*_1_ = 1/*T*_1_, and *R*_2_ = 1/*T*_2_, and the corresponding relaxivities *r*_1_ and *r*_2_ (in units of L mol^−1^ s^−1^) were determined. For graphical presentation the measured relaxation rates of pure water samples have been subtracted.

#### Specific absorption rate (SAR) and intrinsic loss power (ILP)

Room temperature AC magnetometry measurements (hysteresis loops) were carried out using an Advanced AC Hyster magnetometer (Nanotech Solutions, Spain) in the frequency range from 30 kHz to 300 kHz at a magnetic field amplitude of 20 kA m^−1^. The values of AC magnetization were normalized to the iron mass. AC hysteresis loop measurements include three repetitions to obtain average and standard deviation of the magnetic area values. Afterwards, SAR values were calculated according to SAR = *A* × *f*, where *A* is the magnetic area and *f* is the AC magnetic field frequency. For system- and measurement-independent comparison of data, we calculated the intrinsic loss power (ILP) (in units nH m^2^ per g(Fe)) by dividing the SAR value by the frequency and square of the excitation field, ILP = SAR/(*f* × *H*_e_^2^).

#### Magnetic particle imaging

Magnetic Particle Imaging (MPI) measurements of a spiral resolution phantom filled with nanoparticles was acquired using a preclinical 3D-MPI system (Bruker MPI 25/20 FF) working at an excitation frequencie of 25 kHz, an amplitude of 12 mT in three orthogonal dimensions (*x*,*y*,*z*) and a selection gradient of 2.5 T m^−1^ in the *z*-direction and 1.25 T m^−1^ in the *x*- and *y*-directions. Image reconstructions were performed based on the system function (SF) approach using a small (point-like) reference sample measured with identical parameters for all MNP systems. The spiral phantom (2 mm channel width) was filled with 200 μL of sample T2 (30 nm) at an iron concentration of *c*(Fe) = 15 mmol L^−1^. Image reconstruction with a field of view of 28 × 28 × 14 voxels (mm^3^) was performed using 1937 frequency components, 20 Kaczmarz-iterations, and a regularization parameter *λ* = 10^−5^. For comparison, the liver contrast agent and MPI tracer gold standard Resovist® (Meito Sangyo Co. Ltd., Japan) was imaged using identical concentration and parameters.

### Cell culture and determination of cell viability

Immortalized human cerebral microvascular endothelial cells (hCMEC/D3; Biozol) were maintained in rat tail collagen-I (50 μg mL^−1^; Ibidi GmbH) -coated culture flasks in ECBM MV cell culture media (PromoCell) supplemented with 15% fetal bovine serum (FBS), 2.5 ng mL^−1^ basal fibroblast growth factor, 10 μg mL^−1^ sodium heparin (all Sigma-Aldrich).

For nanoparticle treatment, cells were seeded onto 96-well plates coated with rat tail collagen-I (50 μg mL^−1^; Ibidi GmbH) and cultured in the cell culture media for 24 hours. Nanoparticles were diluted in cell culture media and cells were treated starting with a concentration of 500 μM Fe for various time periods. After the treatment, cells were washed with phosphate buffered saline (PBS; Gibco) and a cell-counting-kit (CCK)-8-substrate was added as described by the manufacturer (Sigma-Aldrich). After 60 minutes, the solution was transferred to a new 96-well plate and measured in a microplate reader (VictorX, PerkinElmer) at a wavelength of *λ* = 450 nm. The absorbance of cells treated with the medium was set to 100%.

### Determination of cytotoxicity and apoptosis by flow cytometry

Cells were seeded onto 6-well plates coated with rat-tail collagen-I (50 μg mL^−1^; Ibidi GmbH) and cultured in cell culture media for 24 hours. Nanoparticles were diluted in media and cells were treated with various concentrations of nanoparticles for 24 and 72 hours. After the incubation the cells were washed with PBS (Gibco), detached using trypsin/EDTA solution (Sigma-Aldrich), transferred to a tube and centrifuged at 300*g* for 5 minutes. The cells were stained as described by the manufacturer (bimake), incubated for 15 minutes at room temperature and analyzed by flow cytometry (BD Accuri C6, BD Biosciences). Annexin-V-FITC was detected in the FL-1 channel, while dead (necrotic) cells stained with propidium iodide (PI) were detected in the FL-3 channel. Gating of the cell populations was performed using stained and untreated cells and cells treated with 5% dimethyl sulfoxide (DMSO; Sigma Aldrich) in cell culture media (positive control for apoptosis). 20 000 cells were analyzed for each sample.

## Conclusions

We presented a micromixer synthesis platform for the continuous manufacturing of single-core iron oxide magnetic nanoparticles with well-defined core diameters in the range of 20 to 35 nm, which are stably dispersed in aqueous media. Physicochemical as well as magnetic tuning of particle properties can be achieved by adjusting relevant process parameters, *e.g.* the temperature. High quality particles with core diameters of about 30 nm were found to be optimal for combining imaging application (tracer for MPI and negative contrast agent in MRI) and therapeutic approach (MFH) application.

The micromixer synthesis platform will propel translational research for magnetic nanoparticle based theranostics applications, a further step from basic research to future medicine.

## Conflicts of interest

There are no conflicts to declare.

## Supplementary Material

## References

[cit1] Choi K. Y., Liu G., Lee S., Chen X. (2012). Nanoscale.

[cit2] Cheng C. A., Chen W., Zhang L., Wu H. H., Zink J. I. (2019). J. Am. Chem. Soc..

[cit3] Lammers T., Aime S., Hennink W. E., Storm G., Kiessling F. (2011). Acc. Chem. Res..

[cit4] Wu X. L., Yang H., Yang W. T., Chen X. M., Gao J. X., Gong X. Q., Wang H. J., Duan Y., Wei D. H., Chang J. (2019). J. Mater. Chem. B.

[cit5] Sanson C., Diou O., Thévenot J., Ibarboure E., Soum A., Brûlet A., Miraux S., Thiaudière E., Tan S., Brisson A., Dupuis V., Sandre O., Lecommandoux S. (2011). ACS Nano.

[cit6] Arias J. L., Reddy L. H., Othman M., Gillet B., Desmaële D., Zouhiri F., Dosio F., Gref R., Couvreur P. (2011). ACS Nano.

[cit7] https://clinicaltrials.gov/ct2/show/NCT01411904, 2020

[cit8] Gleich B., Weizenecker R. (2005). Nature.

[cit9] Gleich B. , WO2004091398 A2004091392, 2004

[cit10] Panagiotopoulos N., Duschka R. L., Ahlborg M., Bringout G., Debbeler C., Graeser M., Kaethner C., Ludtke-Buzug K., Medimagh H., Stelzner J., Buzug T. M., Barkhausen J., Vogt F. M., Haegele J. (2015). Int. J. Nanomed..

[cit11] Eberbeck D., Wiekhorst F., Wagner S., Trahms L. (2011). Appl. Phys. Lett..

[cit12] Ferguson R. M., Minard K. R., Krishnan K. M. (2009). J. Magn. Magn. Mater..

[cit13] Ferguson R. M., Khandhar A. P., Kemp S. J., Arami H., Saritas E. U., Croft L. R., Konkle J., Goodwill P. W., Halkola A., Rahmer J., Borgert J., Conolly S. M., Krishnan K. M. (2015). IEEE Trans. Med. Imaging.

[cit14] Thiesen B., Jordan A. (2008). Int. J. Hyperthermia.

[cit15] Chang D., Lim M., Goos J., Qiao R. R., Ng Y. Y., Mansfeld F. M., Jackson M., Davis T. P., Kavallaris M. (2018). Front. Pharmacol..

[cit16] https://clinicaltrials.gov/ct2/show/NCT02033447, 2020

[cit17] Hergt R., Dutz S., Roder M. (2008). J. Phys.: Condens. Matter.

[cit18] Alphandery E., Chebbi I., Guyot F., Durand-Dubief M. (2013). Int. J. Hyperthermia.

[cit19] Alexiou C., Tietze R., Schreiber E., Jurgons R., Richter H., Trahms L., Rahn H., Odenbach S., Lyer S. (2011). J. Magn. Magn. Mater..

[cit20] Al-Jamal W. T., Kostarelos K. (2011). Acc. Chem. Res..

[cit21] Tagami T., Foltz W. D., Ernsting M. J., Lee C. M., Tannock I. F., May J. P., Li S. D. (2011). Biomaterials.

[cit22] Bleul R., Thiermann R., Marten G. U., House M. J., Pierre T. G. S., Hafeli U. O., Maskos M. (2013). Nanoscale.

[cit23] Hufschmid R., Arami H., Ferguson R. M., Gonzales M., Teeman E., Brush L. N., Browning N. D., Krishnan K. M. (2015). Nanoscale.

[cit24] Ali I., Peng C. S., Khan Z. M., Naz I. (2017). J. Basic Microbiol..

[cit25] Baki A., Löwa N., Thiermann R., Bantz C., Maskos M., Wiekhorst F., Bleul R. (2017). International Journal on Magnetic Particle Imaging.

[cit26] Ma J. P., Lee S. M. Y., Yi C. Q., Li C. W. (2017). Lab Chip.

[cit27] Frenz L., El Harrak A., Pauly M., Begin-Colin S., Griffiths A. D., Baret J. C. (2008). Angew. Chem., Int. Ed..

[cit28] Glasgow W., Fellows B., Qi B., Darroudi T., Kitchens C., Ye L. F., Crawford T. M., Mefford O. T. (2016). Particuology.

[cit29] Uson L., Arruebo M., Sebastian V., Santamaria J. (2018). Chem. Eng. J..

[cit30] Pascu O., Marre S., Aymonier C., Roig A. (2013). Nanoscale.

[cit31] Schaifers K. and Voigt H., Landolt-Börnstein: numerical data and functional relationships in science and technology, ed. T. Schmidt-Kaler, 1982

[cit32] Hrens T. J. , Rock physics & phase relations: a handbook of physical constants, American Geophysical Union, 1995

[cit33] Ogrady K., Bradbury A., Popplewell J., Charles S. W., Chantrell R. W. (1985). J. Magn. Magn. Mater..

[cit34] Vanleeuwen D. A., Vanruitenbeek J. M., Dejongh L. J., Ceriotti A., Pacchioni G., Haberlen O. D., Rosch N. (1994). Phys. Rev. Lett..

[cit35] Thomas G., Demoisson F., Chassagnon R., Popova E., Millot N. (2016). Nanotechnology.

[cit36] Teeman E., Shasha C., Evans J. E., Krishnan K. M. (2019). Nanoscale.

[cit37] Ludwig F., Balceris C., Jonasson C., Johansson C. (2017). IEEE Trans. Magn..

[cit38] Poller W. C., Löwa N., Schleicher M., Münster-Wandowski A., Taupitz M., Stangl V., Ludwig A., Wiekhorst F. (2020). Sci. Rep..

[cit39] Kraupner A., Eberbeck D., Heinke D., Uebe R., Schuler D., Briel A. (2017). Nanoscale.

[cit40] Löwa N., Seidel M., Radon P., Wiekhorst F. (2017). J. Magn. Magn. Mater..

[cit41] Wu L. C., Zhang Y., Steinberg G., Qu H., Huang S., Cheng M., Bliss T., Du F., Rao J., Song G., Pisani L., Doyle T., Conolly S., Krishnan K., Grant G., Wintermark M. (2019). Am. J. Neuroradiol..

[cit42] Qiao R. R., Yang C. H., Gao M. Y. (2009). J. Mater. Chem..

[cit43] Modo M. M. j. and Bulte J. W. M., Molecular and Cellular MR Imaging, CRC Press, 2007 10.1162/15353500200505145 16194447

[cit44] Lu Z. G., Deng R. J., Zhen M. M., Li X., Zou T. J., Zhou Y., Guan M. R., Zhang Y., Wang Y. Q., Yu T., Shu C. Y., Wang C. R. (2017). RSC Adv..

[cit45] Hirt A. M., Kumari M., Heinke D., Kraupner A. (2017). Molecules.

[cit46] Laurent S., Dutz S., Häfeli U. O., Mahmoudi M. (2011). Adv. Colloid Interface Sci..

[cit47] Bordelon D. E., Cornejo C., Gruttner C., Westphal F., DeWeese T. L., Ivkov R. (2011). J. Appl. Phys..

[cit48] Kallumadil M., Tada M., Nakagawa T., Abe M., Southern P., Pankhurst Q. A. (2009). J. Magn. Magn. Mater..

